# A Potential Anticancer Mechanism of Finger Root (*Boesenbergia rotunda*) Extracts against a Breast Cancer Cell Line

**DOI:** 10.1155/2022/9130252

**Published:** 2022-09-05

**Authors:** Muhammad Hermawan Widyananda, Septian Tri Wicaksono, Kurnia Rahmawati, Sapti Puspitarini, Siti Mariyah Ulfa, Yoga Dwi Jatmiko, Masruri Masruri, Nashi Widodo

**Affiliations:** ^1^Biology Department, Faculty of Mathematics and Natural Sciences, Brawijaya University, Malang, Indonesia; ^2^Agricultural Product Technology, Faculty of Agricultural Technology, Brawijaya University, Malang, Indonesia; ^3^Chemistry Department, Faculty of Mathematics and Natural Sciences, Brawijaya University, Malang, Indonesia

## Abstract

Breast cancer is the most common type of cancer women suffer from worldwide in 2020 and the 4th leading cause of cancer death. *Boesenbergia rotunda* is an herb with high potential as an anticancer agent. This study explores the potential bioactive compounds in *B. rotunda* as anti-breast cancer agents using *in silico* and *in vitro* approaches. The *in silico* study was used for active compound analysis, selection of anticancer compound candidates, prediction of target protein, functional annotation, molecular docking, and molecular dynamics simulation, respectively. The *in vitro* study was conducted by measurement toxicity, rhodamine 123, and apoptosis assays on T47D cells. Based on the KNApSAcK database, *B. rotunda* contained 20 metabolites, which are dominated by chalcone and flavonoid groups. Seven of them were predicted to have anticancer activity, namely, sakuranetin, cardamonin, alpinetin, 2S-pinocembrin, 7.4′-dihydroxy-5-methoxyflavanone, 5.6-dehydrokawain, and pinostrobin chalcone. These compounds targeted proteins related to cancer progression pathways such as the PI3K/Akt, FOXO, JAK/STAT, and estrogen signaling pathways. Therefore, these compounds are predicted to inhibit growth and induce apoptosis of cancer cells through their interactions with MMP12, MMP13, CDK4, JAK3, VEGFR1, VEGFR2, and KCNA3. Anticancer activity of *B. rotunda* through *in vitro* study confirmed that *B. rotunda* extract is strong cytotoxic and induces apoptosis of breast cancer cell lines. This study concludes that *Boesenbergia rotunda* has potency as an anticancer candidate.

## 1. Introduction

Breast cancer is the most common type of cancer in women worldwide in 2020, followed by lung, prostate, and skin cancers. This type of cancer is the 4th leading cause of death from cancer after lung, stomach, and liver cancers [1]. Breast cancer patients are mostly in the reproductive age, namely, 30 to 39 years [2]. Previous studies have reported that the breast cancer deaths were increasing over the last 25 years. Thus, the effort is needed for the discovery of cancer drugs that are effective in treating breast cancer [3]. Breast cancer is mostly caused by obesity, alcohol consumption, genetics, and age [4]. The breast cancer cells caused by alteration of specific genes that result in dysregulation of several pathways related to cell proliferation and survival [5].

Several pathways were dysregulated in breast tumor cells, including the estrogen signaling pathway, PI3K/Akt signaling pathway, and JAK/STAT signaling pathway. The estrogen signaling pathway that plays a role in regulating cell division is dysregulated through overactivity of *estrogen receptor alpha* (ER*α*). ER*α* will be activated after binding to estrogen, form a dimer, and then attach to the estrogen response element (ERE) in DNA [6]. ERE consists of genes related to cell growth [7, 8]. The PI3K/Akt signaling pathway has a crucial role in the progression of breast cancer cells because it is involved in proliferation, survival, invasion, migration, apoptosis, glucose metabolism, and DNA repair in cells. The mutation of PI3K protein, especially in ER + subtype breast cancer, causes PI3K hyperactivation [9]. Several protein tyrosine kinase receptors of the PI3K/Akt pathway, such as HER2 and EGFR, are overexpressed and mutated in breast cancer cells [10, 11]. The JAK/STAT signaling pathway has an essential role in the development of breast cancer cells. Three major proteins play a role in this pathway including receptor tyrosine kinase, JAK (Janus kinase), and STAT (signal transducer and activator of transcription). Alteration of these proteins causes proliferation and metastasis in breast cancer cells [12]. Various therapies have been developed to inhibit the activity of these pathways from preventing breast cancer progression.

Today, the most common treatment for breast cancer is chemotherapy [13]. However, chemotherapy has adverse side effects for patients such as constipation, dyspnea, fatigue, pain, rash, vomiting, and pain, and the most dangerous side effect is peripheral neuropathy [14, 15]. Cisplatin is the main chemotherapy drug for treating solid tumors; however, it has side effects that caused kidney and liver damage [16]. Doxorubicin is a DNA intercalation agent that effectively inhibits tumor progression. Unfortunately, doxorubicin has a cardiotoxicity effect [17]. Gefitinib is quite popularly used for cancer treatment that targets the epidermal growth factor receptor (EGFR). However, this drug has side effects such as rash, diarrhea, and even inflammation of the lower urinary tract and bladder [18]. Other chemotherapy drugs also have side effects that are no less dangerous. In addition, chemotherapy is not provided in all hospitals globally because it is expensive. Therefore, agents for cancer therapy are needed from natural sources that are cheap and have minimal side effects.

*Boesenbergia rotunda* is an herb with high potential as an anticancer agent. *B. rotunda* belongs to the Zingiberaceae family that grows in Southeast Asia, India, Sri Lanka, and southern China. In these countries, *B. rotunda* is known as a medicinal plant that can treat various diseases [19]. Previous research mentioned that *B. rotunda* hexane extract was toxic to liver, lung, and colon cancer cell lines [20]. Previous studies stated that the ethanol extract of *B. rotunda* has antiproliferative activity and induces apoptosis in HeLa cervical cancer cells [21]. The anticancer activity of *B. rotunda* is predicted because of the presence of their bioactive compounds. Sakuranetin has a cytotoxic effect on B16BL6 melanoma cells by inhibiting the PI3K/Akt signaling pathway [22]. Cardamonin and pinostrobin chaconne isolated from rhizome *B. rotunda* have a cytotoxic effect on the H-29 colon cancer cell line [23]. No previous studies have investigated the potential anticancer activity of all potential bioactive compounds found in *B. rotunda*. *In silico* followed by *in vitro* experiments are the most appropriate approach for the initial study of anticancer potential bioactive compounds present in *B. rotunda*.

The *in silico* approach is very appropriately used for drug candidate screening because it can accelerate the finding of drug candidate compounds by predicting their cellular and molecular mechanisms [24]. The results will be more valid if supported by an *in vitro* approach, and there is a strong positive correlation between *in silico* and *in vitro* results, [25, 26]. Therefore, *in silico* and *in vitro* approaches are very appropriate for this study. This study aimed to explore the potential bioactive compounds in *B. rotunda* as anti-breast cancer agents using *in silico* and *in vitro* approaches.

## 2. Methods

### 2.1. Compound Data Mining

The compounds contained in *B. rotunda* were obtained from the KNApSAcK database (https://www.knapsackfamily.com/KNApSAcK/) and previous studies. The KNApSAcK is a plant metabolite database containing 20,741 species and 50,048 metabolites [27]. Canonical SMILES of all compounds were obtained from the PubChem database (https://pubchem.ncbi.nlm.nih.gov/).

### 2.2. Screening Based on Druglikeness and Probable Activity

The compounds contained in *B. rotunda* obtained from the database were selected by screening using druglikeness and possible bioactivity. Druglikeness screening was conducted using the SWISS ADME web server (https://www.swissadme.ch/) to identify compounds that might have medicinal properties that integrated with those of the Lipinski, Ghose, Veber, Egan, and Muegge rules. Screening for possible activities was conducted to select the compounds that have function to interact with pathway signaling in the cells using the PASS Online web server (https://www.way2drug.com/passonline/). The pathway activity was selected based on their functions as anti-breast cancer agents such as MMP9 expression inhibitor [28], apoptosis agonist [29], JAK2 expression inhibitor [30], antineoplastic (breast cancer) [31] and proliferative disease treatment agent [32], caspase-3 stimulant [33], caspase-8 stimulant [33], topoisomerase I inhibitor [34], topoisomerase II inhibitor [34], cancer-associated disorder treatment agent [35], protein kinase C inhibitor [36], CDC25 phosphatase inhibitor [37], and CDK9/cyclin T1 inhibitor [38].

### 2.3. Protein Target Prediction

The compounds that met the druglikeness and probable activity parameters were used for target protein prediction. Direct targets were predicted using the SWISS Target Prediction database (https://www.swisstargetprediction.ch/); then, the five proteins that related to breast cancer were taken. SwissTargetPrediction is a web server to accurately predict the target protein based on the similarity of compound structure to a previously known compound [39]. Indirect target proteins were obtained from direct targets using the STRING 11.0 database with a confidence level of 0.4 and a maximum interaction number of 5. STRING is a database that predicts protein-protein interactions computationally [37]. Visualization of the target protein analysis was performed using Cytoscape 3.8.2.

### 2.4. Functional Annotation

Functional annotation was performed to predict the role of target proteins in cell biology systems using the Database for Annotation, Visualization, and Integrated Discovery web server (https://david.ncifcrf.gov/) [40]. The databases used for this analysis were the Gene Ontology (GO) and the Kyoto Encyclopedia of Genes and Genomes (KEGG) pathway databases. The GO database groups genes based on their roles in cells according to three domains, namely, molecular function, biological process, and cellular component [41]. The KEGG pathway is a database that groups genes based on cellular pathways [42].

### 2.5. Molecular Docking

Molecular docking was conducted between the compound and its direct target protein. The protein's three-dimensional structure was obtained from the PDB RCSB database (https://www.rcsb.org/). The water molecules and contaminant ligands were removed using the Biovia Discovery Studio 2019 software (Dassault Systèmes Biovia, San Diego, CA, USA). The three-dimensional structure of the compound contained in *B. rotunda* was obtained from the PubChem database and then was prepared using OpenBabel [43] integrated into the PyRx software. Specific docking was conducted between compounds on the active site of each protein using the AutoDock Vina software integrated into PyRx 0.8 [44, 45]. The docking results were visualized using the Biovia Discovery Studio 2019 software.

### 2.6. Molecular Dynamics Simulation

Molecular dynamics simulation was conducted using the YASARA (Yet Another Scientific Artificial Reality Application) software with the AMBER14 force field [46]. The system conditions were adjusted according to the physiological conditions of the cells (37°C, pH 7.4, 1 atm, and 0.9% salt content) for 20 ns. The macro programs used were md_run to run simulations, md_analyze to analyze RMSD, and md_bindingenergy to analyze the molecular dynamics binding energy of protein-ligand complexes.

### 2.7. *B. rotunda* Extraction

Six grams of powdered *B. rotunda* (Materia Medika, Batu, East Java, Indonesia) and distilled water or 96% ethanol in a ratio of 1 : 10 were put in a vessel of MAE (microwave-assisted extraction) (Anton-Paar). MAE was operated according to the specified protocol (holding temperature, 50°C; 5 min warming up, 50°C; time holding, 10 min; 5 min cooling down; power, 1500 W). The extract was filtered using the Whatman filter paper and then evaporated using a Buchi R-210 Rotavapor System (50 rpm, 37°C). The obtained extract was stored at 4°C.

### 2.8. Total Phenol and Flavonoid Analysis

Total phenol analysis was conducted using the Folin–Ciocalteu method with gallic acid as standard. This assay adopted the method of Jing et al. [47]. A total of 100 *μ*L of *B. rotunda* extract and standard solution (1.5625–100 *μ*g/mL) were added to 1.0 mL of Folin–Ciocalteu reagent, which had been diluted 10 times with distilled water. The solution was added with 1 mL of Na_2_CO_3_ (7.5%, w/v) and incubated in the dark for 90 min at room temperature. Total phenolic content was measured using spectrophotometry at a wavelength of 725 nm. This test was performed in triplicate. Total flavonoid content is expressed in terms of gallic acid equivalent (mgGE/g).

Total flavonoid analysis was conducted using the aluminum chloride colorimetric assay method adopted from Chtatatikun and Chiabchalard [48] and Novia Sembiring et al. [49] with modifications. The standard solution used was quercetin in 96% ethanol. *B. rotunda* extract (1 mg/mL) and 50 *μ*L standard (1.5625–100 *μ*g/mL) were added to 10 *μ*L AlCl_3_ (10%, w/v), followed by the addition of 150 *μ*L 96% ethanol. The 10 *μ*L of 1 M CH_3_COONa was added to the solution. The mixture was added to 10 *μ*L of 1 M CH3COONa. A 96% ethanol solution was used as a blank. The mixture was incubated for 40 min at room temperature in the dark. Absorbance was measured at a wavelength of 405 nm. This test was performed in triplicate. Total flavonoid content is presented in terms of quercetin equivalent (mgQE/g).

### 2.9. DPPH and NO Scavenging Assay

The antioxidant activity of the ethanol extract of *B. rotunda* was analyzed using the 2,2-diphenyl-1-picrylhydrazyl (DPPH) assay and ascorbic acid as the standard. One hundred microliters of extract and standard at a concentration of 31.25 to 1000 *μ*g/mL were added to 100 *μ*L of 0.4 M DPPH solution on a 96-well plate. The mixture was incubated at room temperature for 30 min. Absorbance readings were conducted at a wavelength of 490 nm using a ELx880TM microplate reader (BioTek Instrument, USA). This assay was performed in triplicate. The antioxidant activity of the extracts was determined based on the IC_50_ value.

The antioxidant activity through NO scavenging was analyzed using the NO scavenging assay method with some optimizations [50]. A total of 60 *μ*L of the extract was serially diluted onto a 96-well plate. A total of 60 *μ*L sodium nitroprusside (SNP) at a concentration of 10 mM was added to each well. The control used was SNP only. The SNP was dissolved in phosphate-buffered saline and then incubated at room temperature under bright lighting for 150 min. Griess reagent (5% phosphoric acid, 1% sulfanilamide, and 0.1% naphthyl ethylene diamine dihydrochloride) was added to each well. Absorption readings were conducted at a wavelength of 570 nm using a ELx880TM microplate reader. This assay was performed in three replicates. The NO scavenging activity of the extract was determined based on the IC_50_ value.

### 2.10. Cell Culture Preparation

The breast cancer cell line T47D and the human fibroblast cell line TIG-1 were obtained from the Animal Physiology, Structure, and Growth Laboratory, Brawijaya University. The cells were cultured using complete media (RPMI 1640 (Gibco, USA) for T47D and MEM (Gibco, USA) for TIG-1 + 10% fetal bovine serum (Gibco, USA) + 1% penicillin-streptomycin (Gibco, USA)) on 60 mm dishes. Cells were incubated at 37°C and 5% CO_2_.

### 2.11. Cell Viability Assay

The T47D and TIG-1 cell lines were seeded onto 96-well plates at a density of 7,500 cells per well and incubated at 37°C and 5% CO_2_ for 24 h. The cells were treated with aqueous and ethanol extracts of *B. rotunda* at concentrations of 0, 10, 20, and 40 *μ*g/mL for 24 h. The treatment medium was replaced with a medium containing 5% WST-1 (Sigma-Aldrich, USA), and the cells were incubated for 30 min. The absorbance was measured at a wavelength of 450 nm using an ELx880TM absorbance microplate reader. The IC_50_ value was determined using the inhibition curve. The assay was conducted in triplicate [51].

### 2.12. Apoptosis Assay

T47D cells were seeded onto 24-well plates (75,000 cells/well) and then incubated for 24 h. The cells were then treated with various doses of ethanol extract of *B. rotunda*, 0 (untreated), 20, 40, and 80 *μ*g/mL, for 24 h. The cells were harvested and added with annexin V and propidium iodide (PI) (BioLegend, USA) and incubated in the dark at 4°C for 20 min. The cell suspensions were run using the flow cytometer (BD FACSCalibur, USA), and the data analysis was performed using the CellQuest software (BD Bioscience, USA). The analysis was conducted in triplicate [51].

### 2.13. Rhodamine Assay

The rhodamine assay referred to the method of Wei et al. [52] with some modifications. T47D cells were seeded onto 24-well plates at a density of 75,000 cells/well and then incubated at 37°C and 5% CO_2_ for 24 h. The cells were treated with *B. rotunda* ethanol extract at the concentrations of 0 (untreated), 20, 40, and 80 *μ*g/mL and incubated for 24 h. After incubation, 2 *μ*M rhodamine 123 (Thermo Fisher Scientific, USA) was added to each well and the cells were incubated for 1 h at 37°C and 5% CO_2_. The cells were harvested and centrifuged at 2500 rpm and 10°C for 5 min. The pellet was resuspended with basal media, incubated for 30 min at room temperature, and then washed with phosphate-buffered saline. The rhodamine 123 staining analysis was conducted using the FACSCalibur analyzer, and the data were obtained using the CellQuest software. The assay was conducted in triplicate.

### 2.14. Statistical Analysis

Statistical analysis for rhodamine and apoptosis test results was performed using a one-way analysis of variance with Tukey's HSD as a post hoc test (*p* < 0.01). Analyses were performed using the SPSS software version 23 (SPSS, Inc., Chicago, IL, USA).

## 3. Results

### 3.1. Bioactive Compounds Contained in *B. rotunda* Based on Database

**Figure 1:**
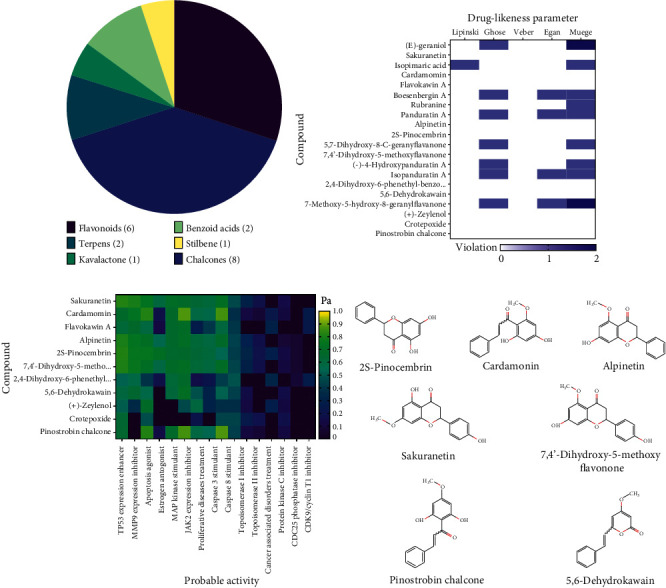
Bioactive compounds contained in *B. rotunda*. (a) Group of bioactive compounds contained in *B. rotunda* based on the KNApSAcK database. (b) Druglikeness screening. (c) PASS online screening. (d) The seven compounds that met the druglikeness and probable activity parameters.

Based on the KNApSAcK database, there are 20 bioactive compounds in *B. rotunda*. The compounds contained in *B. rotunda* are dominated by chalcone and flavonoids. The chalcone compounds in *B. rotunda* are cardamonin, flavokawin A, boesenbergin A, rubranine, panduratin A, (-)-4-hydroxypanduratin A, isopanduratin A, and pinostrobin chalcone. The flavonoid compounds are sakuranetin, 7-methoxy-5-hydroxy-8-geranylflavanone, alpinetin, 2S-pinocembrin, 5,7-dihydroxy-8-C-geranylflavanone, and 7,4′-dihydroxy-5-methoxyflavanone. The remainder are two terpenes ((E)-geraniol and isopimaric acid), one kavalactone (5,6-dehydrokawain), two benzoic acids ((+)-zeylenol and crotepoxide), and one stilbenes (2,4-dihydroxy-phenethyl-benzoic acid methyl ester) (Table 1 & Figure 1(a)).

### 3.2. Screening Based on Druglikeness and Probable Activity

Druglikeness screening aimed to select compounds that have drug-like characteristics. Of the 20 compounds, 11 compounds met the characteristics of medicinal compounds, namely, sakuranetin, cardamonin, flavokawin A, alpinetin, 2S-pinocembrin, 7,4′-dihydroxy-5-methoxyflavanone, 2,4-dihydroxy-6-phenethyl-benzoic acid methyl ester, 5,6-dihydrokawain, (+)-zeylenol, crotepoxide, and pinostrobin chalcone (Figure 1(b)). The eleven compounds were continued to screen for probable bioactivity to predict the possibility of their activity in inhibiting breast cancer progression (Figure 1(c)). In this screening, compounds with a Pa value of more than 0.7 were selected. A Pa value of more than 0.7 indicates the compound has a high potential for carrying out related activities [59]. Of the 11 compounds, 7 compounds had a Pa value always more than 0.7, namely, sakuranetin, cardamonin, alpinetin, 2S-pinocembrin, 7.4′-dihydroxy-5-methoxyflavanone, 5.6-dehydrokawain, and pinostrobin chalcone (Figure 1(d)).

### 3.3. Protein Target Prediction

**Figure 2:**
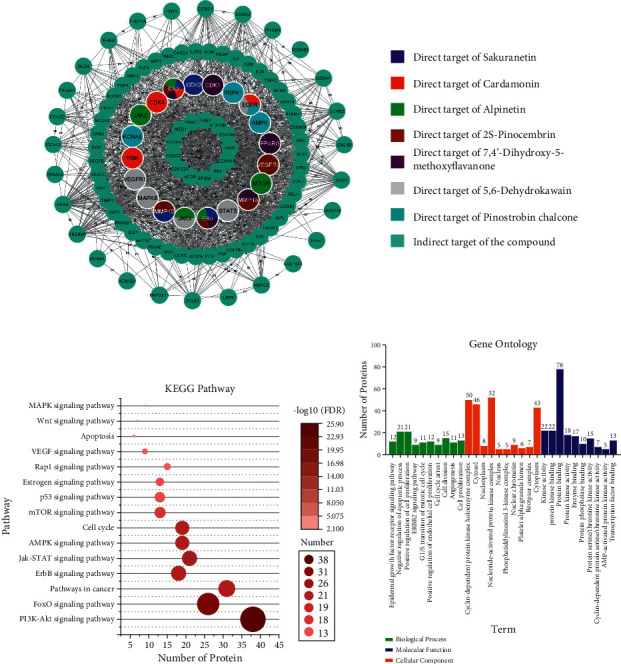
Target proteins of the seven chosen compounds in *B. rotunda*. (a) Direct and indirect targets of the compounds. (b) KEGG pathway related to target proteins. (c) GO terms related to the target proteins.

The protein target prediction results showed a total of 107 target proteins consisting of 20 direct and 87 indirect targets (Figure 2(a)). The direct targets of sakuranetin are CDK2, ER*α*, ER*β*, and MMP12. The direct targets of cardamonin are EGFR, ER*β*, CDK4, and PI3K. Alpinetin's direct targets are MTOR, ER*α*, ERβ, JAK2, and JAK3. The direct targets of 2S-pinocembrin are VEGFR2, MMP13, ER*α*, MMP12, and ERβ. The direct targets of 7,4′-dihydroxy-5-methoxyflavanone are CDK1, PPARG, MMP13, ER*α*, and ERβ. The direct targets of 5,6-dehydrokawain are EGFR, STAT3, JAK3, MAPK8, and VEGFR1. The direct targets of pinostrobin chalcone are EGFR, AMPK, KCNA3, and PDPK1. Meanwhile, the indirect target is shown in Figure 2(a).

### 3.4. Functional Annotation

All target proteins have roles in cancer cell progression as shown in Figure 2. Based on the GO analysis, the target proteins have roles in cancer-related biological processes such as epidermal growth factor receptor signaling pathway, negative regulation of apoptosis, and positive regulation of cell proliferation, among others. All target proteins are mostly located in the cytoplasm, nucleoplasm, and membrane. These proteins functioned in kinase activity, protein kinase binding, protein binding, enzyme binding, and so on (Figure 2(c)). Based on the KEGG pathway, these target proteins play a role in cancer-related pathways such as the PI3K/Akt, FOXO, ErbB, and JAK/STAT signaling pathways (Figure 2(b)). The results of this functional annotation analysis showed that based on the GO and KEGG pathway databases, the target proteins have a cancer-related role.

### 3.5. Molecular Docking

**Figure 3:**
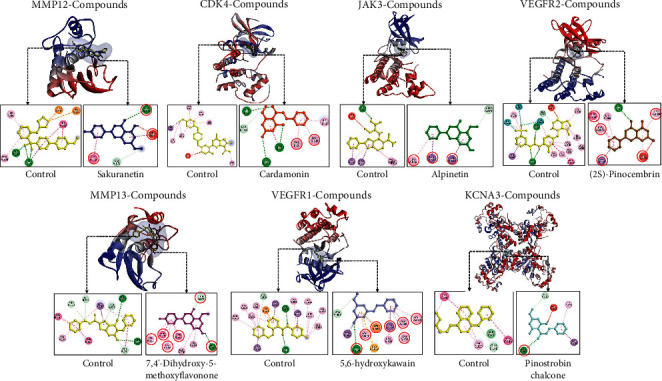
Molecular docking simulation results. The red circle indicates the same residue as the control.

Molecular docking simulation was conducted on the seven bioactive compounds with direct target proteins, and the results are shown in Table 2. The most negative binding affinity value of docking results was selected for further molecular dynamics simulation. The docking results showed that the seven bioactive compounds contained in *B. rotunda* bind to their respective target proteins on the same site as the control. This indicates that the compound is predicted to have similar activity to the control. The most negative binding affinity values of docking results are shown in Figure 3. Sakuranetin binds to the active site of MMP12 by forming 2 hydrogen bonds and 2 hydrophobic bonds. Sakuranetin had the same residues as the control, namely, in Ala182, Leu181, and His218. Cardamonin bound to the active site of CDK4 by forming 4 hydrogen bonds and 4 hydrophobic interactions and bound to the same residue as the control, namely, to Val20 and Leu147. Alpinetin formed 1 hydrogen bond and 3 hydrophobic interactions with JAK3. Alpinetin bound to the same residue as the control, namely, to Leu956, Leu828, and Val836.2S-Pinocembrin bound to the active site of VEGFR2 by forming 1 hydrogen bond and 6 hydrophobic interactions. This compound formed bonds at the same residue as the control, namely, at Leu840, Val848, Phe104, Leu1035, and Cys1045. 7,4′-Hydroxy-5-methoxyflavanone binds to the active site of MMP13 by forming 2 hydrogens and 6 hydrophobic interactions. This compound contains the same residues as the control, namely, Thr224, Met232, Tyr223, Leu197, His201, and Val198. 5,6-Dehydrokawain bound to the VEGFR1 active site by forming 1 hydrogen and 9 hydrophobic interactions. 5,6-Dehydrokawain binds to the same residues as the control, namely, to Val841, Ala859, Cys912, Leu1029, Lys861, Val909, and Asp1040. Pinostrobin chalcone binds to KCNA3 by forming 2 hydrogens and 2 hydrophobic interactions. This compound only binds to the same residue as the control, namely, to Glu168.

### 3.6. Molecular Dynamics Simulation

**Figure 4:**
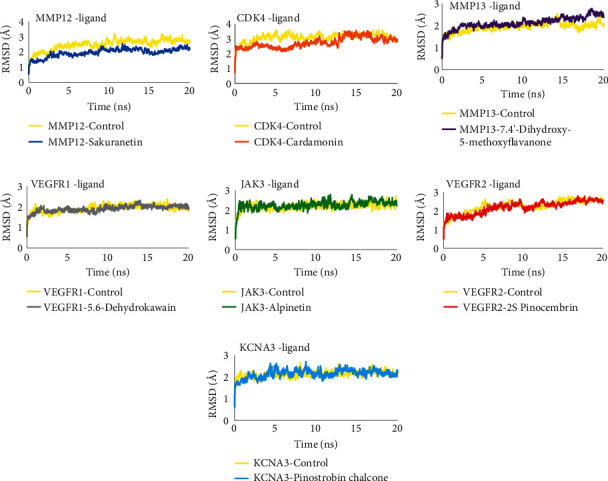
Root mean square deviation (RMSD) of the protein-compound complex.

**Figure 5:**
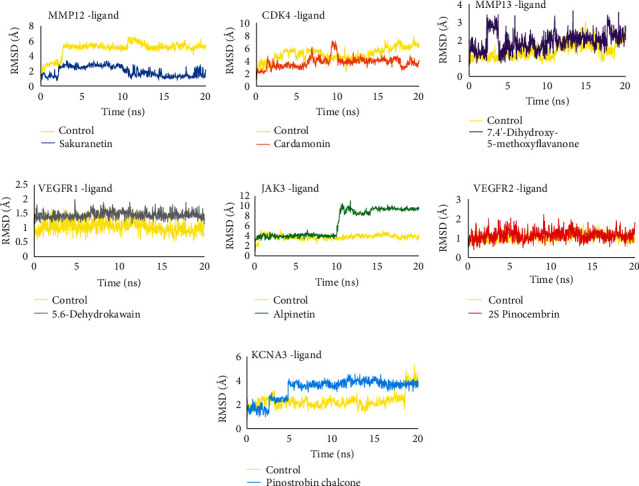
RMSD ligand movement of the protein-compound complex.

**Figure 6:**
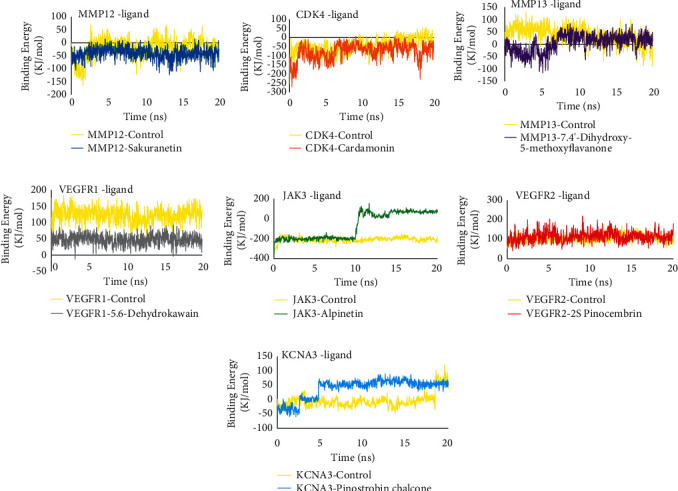
Molecular dynamics binding energy of each complex.

Molecular dynamics simulations were performed to analyze the stability of the interaction between proteins and *B. rotunda* compounds. The parameters used in this simulation are protein-ligand complex RMSD, ligand movement RMSD, and molecular dynamics binding energy. The protein-ligand complex RMSD represents the stability of the complex during a 20 ns simulation. The complex RMSD results showed that all compounds had stable values similar to the control and all complexes had RMSD values below 3 Å, which means they are stable [60, 61] (Figure 4). Ligand movement RMSD represents the stability of ligands when interacting with proteins. The ligand is in a stable state if it does not move much during the simulation, which is indicated by a stable ligand movement RMSD value. The results showed that almost all compounds in *B. rotunda* had stable ligand movement RMSD similar to the control. Sakuranetin has a more stable RMSD value than the control. Ligand movement RMSD of alpinetin upon binding to JAK3 increased at ∼10 ns but stabilized from ∼12 ns until the end of the simulation (Figure 5). Molecular dynamics binding energy also represents the stability of the protein-ligand interaction; the more positive the binding energy value, the more stable the protein-ligand interaction [62]. Overall, the molecular dynamics binding energy results showed that all protein complexes in *B. rotunda* were stable. Complexes of CDK4-cardamonin, VEGFR1-5,6-dyhidrokawain, VEGFR2-2S-pinocembrin, and KCNA3-pinostrobin chalcone tend to be stable because their binding energy values do not fluctuate much, but their stability is still below the control protein complex. The MMP12-sakuranetin and MMP13-7.4′-dihydroxy-5-methoxyflavanone complexes have high interaction stability because their binding energy is almost the same as that of the control. The JAK3-alpinetin complex is very stable because it has a more positive binding affinity value than the control (Figure 6). Overall, molecular dynamics simulations showed that the interactions between proteins and compounds contained in *B. rotunda* are stable and that these compounds have a high potential to act as inhibitors of related proteins.

### 3.7. Total Phenol and Flavonoid of *B. rotunda* Extracts

**Figure 7:**
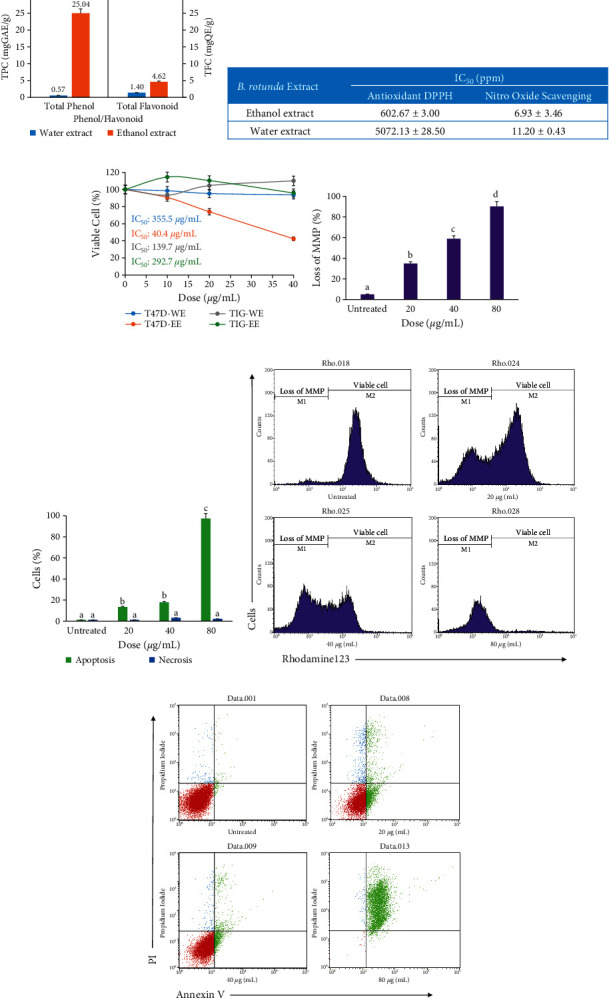
Anticancer activity of *B. rotunda* extract. (a) Total phenol and flavonoid water and ethanol extracts of *B. rotunda*. (b) IC_50_ values for DPPH and NO scavenging tests on aqueous and ethanol extracts of *B. rotunda*. (c) Toxicity test of aqueous and ethanol extracts of *B. rotunda* on the cell line T47D. (d) and (f) Rhodamine 123 test results. (e) and (g) Apoptotic test results with annexin V and PI. Different letters in bar indicate significant difference at *p* <  0.01. Each value represents the average of three experiments.

Total phenol and flavonoid assays were conducted to predict the presence of potentially bioactive compounds (based on *in silico* results) in the extracts used because most of these potential compounds belong to the phenolic and flavonoid groups. The results showed differences in total phenols and flavonoids in the aqueous and ethanol extracts of *B. rotunda*. Total phenol in the ethanol extract was much higher (25.04 mg GAE/g) than in the aqueous extract (0.57 mg·GAE/g). The total flavonoid in the ethanol extract was also higher (4.52 mg·QE/g) than in the aqueous extract (1.40 mg·QE/g) (Figure 7(a)). These results indicate that phenolic and flavonoid compounds that have potential as anti-breast cancer agents are predicted to be more abundant in the ethanol extract of *B. rotunda*.

### 3.8. Antioxidant Activity of *B. rotunda* Extracts

The antioxidant assay results confirmed the total phenol and flavonoid results. The results of the antioxidant activity test using the DPPH and NO scavenging assay showed that the ethanol extract of *B. rotunda* had better antioxidant activity than the aqueous extract. The IC_50_ value of the DPPH test for the ethanol extract was 602 ± 3.00 ppm, while that of the water extract was 5072.13 ± 28.5 ppm. The NO scavenging activity of the ethanol extract was also higher than that of the aqueous extract. The ethanol extract had an IC_50_ value of 6.93 ± 3.46 ppm, while that of the water extract was 11.20 ± 0.43 ppm. These results indicate that the ethanol extract of *B. rotunda* has more antioxidant compounds than the water extract (Figure 7(b)).

### 3.9. Toxicity of the Extracts to the T47D Breast Cancer Cell Line

The toxicity assay aimed to analyze the toxic effect of the extract on T47D cells, and the results are shown in Figure 7(c). The IC_50_ value determines this toxic effect. The results showed that the ethanol extract of *B. rotunda* was much more toxic to T47D cells with an IC_50_ value of 40.4 *μ*g/mL than the aqueous extracts with an IC_50_ value of 355.5 *μ*g/mL. Both aqueous and ethanolic extracts of *B. rotunda* were not toxic to TIG-1 cells, characterized by a high IC_50_ value. From the results of this toxicity test, it can be determined that the ethanol extract of 40.4 *μ*g/mL *B. rotunda* can reduce the viability of the T47D cell population by half and is specific for cancer cells. However, it is not known whether the cell death caused by this extract is necrotic or apoptotic. Rhodamine and apoptosis tests are necessary to determine the type of cell death.

### 3.10. Ethanol Extract of *B. rotunda* Induced Loss of MMP in T47D Cells

The effect of the ethanol extract of *B. rotunda* on the mitochondrial membrane potential (MMP) was evaluated using rhodamine 123 (Figures 7(d) and 7(f)). Rhodamine 123 is a green fluorescent dye used to stain mitochondria with MMP, which indicates that the cells are viable. Cells are deprived of MMP that were not get stained by rhodamine 123, indicating that the cells are not viable [63]. The results showed that the higher the dose of *B. rotunda* ethanol extract, the more cells lost MMP. The number of cells that lost MMP increased significantly with the increasing dose of the extract.

### 3.11. Ethanol Extract of *B. rotunda* Induced Apoptosis of T47D Cells

The effect of apoptotic induction of *B. rotunda* ethanol extract on T47D cells was evaluated using annexin V and PI (Figures 7(e) and 7(g)). Annexin V detects apoptosis by binding to phosphatidylserine, which is exposed to extracellular sites when cells undergo apoptosis and PI detects necrosis by binding to DNA [64]. The results showed that the number of T47D cells undergoing apoptosis increased with increasing extract dose, although it was not significant between doses of 20 and 40 *μ*g/mL. The number of necrotic cells was not significantly different between the control and the treatment, which indicated that the extract did not significantly cause necrosis in T47D cells.

## 4. Discussion

Based on the KNApSAcK database, *B. rotunda* contains bioactive compounds, which are dominated by phenol group compounds, namely, chalcone and flavonoids (Figure 1(a)). These phenolic compounds are predicted to have anti-breast cancer effects. The content of phenolic compounds and flavonoids in the ethanol extract of *B. rotunda* was higher than that of the aqueous extract (Figure 7(a)). Therefore, the compounds contained in *B. rotunda* obtained from the database are most likely present in the ethanol extract. These results are supported by antioxidant tests using DPPH and NO scavenging assays, where ethanol extract has higher antioxidant activity than aqueous extract (Figure 7(b)). This higher antioxidant activity is most likely caused by the more significant number of phenolic compounds present in the ethanol extract. Compounds in *B. rotunda* with high antioxidant activity are pinostrobin chalcone, alpinetin, and cardamonin [65, 66]. This result is in line with that in previous studies, which state that extraction with ethanol solvent can obtain more phenolic compounds than extraction with water solvent. [67].

Drug likeness and bioactivity pathway prediction were used to select the active compounds of *B. rotunda* that have potential as anticancer agents. Seven compounds selected are sakuranetin, cardamonin, alpinetin, 2S-pinocembrin 7.4′-dihydroxy-5-methoxyflavanone, 5.6-dehydrokawain, and pinostrobin chalcone. Some of these bioactive compounds have been known to have anticancer activity in previous studies, but the molecular mechanism is partly unknown. Sakuranetin isolated from *Artemisia dracunculus* inhibits the proliferation of esophageal squamous cell carcinoma cells via induction of DNA damage and mitochondrial membrane potential loss [68]. Cardamonin and alpinetin can suppress proliferation and induce apoptosis of prostate and ovarian cancer cells by modulating the STAT3 pathway [69, 70]. Pinostrobin chalcone and 5,6-dehydrokawain have antiproliferative effects on various cancer cell lines [71, 72]. Research on the anticancer effects of 2S-pinocembrin and 7.4′-dihydroxy-5-methoxyflavanone is still very limited. In addition, the combination of these compounds in inhibiting the growth of breast cancer has not been explained. This study describes how these seven potential compounds work together to provide an anti-breast cancer effect.

Direct and indirect target proteins of the seven compounds contained in *B. rotunda* were closely related to breast cancer progression. These proteins have a role in signaling pathways related to breast cancer. The PI3K/Akt and mTOR signaling pathways are activated by receptor tyrosine kinases that lead to tumor cell growth and proliferation [73]. The FOXO signaling pathway plays a role in tumor suppression. The FOXO protein regulates the expression of genes important for tumor cell growth, such as p27, CDKN1B, TNFSF10, and GADD45 [74]. The JAK/STAT signaling pathway is activated by receptor tyrosine kinases such as EGFR and interleukin receptor, which activate the STAT3 protein. STAT3 is a transcription factor for breast cancer proliferation-associated genes such as *CCND1, c-myc*, *BCL2*, and *BAX* [75]. ER*α* and ER*β* are involved in the estrogen signaling pathway. ER*α* and ER*β* are activated after binding to estrogen, form dimers, and bind to target genes such as *CCND1*, *HIF1A*, and *IL6*, which have a role in breast cancer proliferation [7]. The Wnt signaling pathway is activated when the Wnt ligand binds to the LRP and Frizzled protein complex, thereby activating catenin, which in turn regulates transcription of *c-myc*, *CCND1*, *MMP7*, and *CD44* [76]. The MAPK pathway enhances the sensitivity of breast cancer cells to estradiol so that cells grow faster [77]. The VEGF signaling pathway induces angiogenesis in breast cancer [78]. The results of the functional annotation with the KEGG pathway are in line with those of the GO analysis. The interaction between the compounds contained in *B. rotunda* and the target proteins will potentially affect these pathways.

The interaction between sakuranetin and MMP12 has a low binding affinity value. In addition, the interaction between and 7.4′-dihydroxy-5-methoxyflavanone and MMP13 also has a low binding affinity value. Interestingly, theses interactions have stability as same as the control. Therefore, the two compounds were predicted to be high in inhibiting the activity of MMP12 and MMP13 proteins, respectively. MMPs have a role as metastasis-promoting enzymes by degrading all extracellular matrix proteins [79]. MMP12 was highly expressed in various tumor cell comparisons with normal epithelial cells and positively correlated with cancer cell invasion [80]. MMP12 inactivation could inhibit lung adenocarcinoma cells' growth, invasion, and metastasis [81, 82]. MMP13 has significantly increased expression in breast cancer tissue and is predicted to play a significant role in tumor invasion and metastasis [83]. Previous studies have shown that inhibition of MMP13 activity causes inhibition of the growth of the breast cancer cell lines MDA-MB-231 and 4T1.2 [84]. Based on this explanation, it was stated that the inhibition of the MMP12 and MMP13 activities correlated with the inhibition of cancer cell growth. This was confirmed in this study, which showed that the higher the dose of *B. rotunda* extract, the lower the number of cells (Figure 7(c)). However, the molecular mechanism related to apoptosis caused by MMP12 and MMP13 inhibition needs further research.

Angiogenesis-related proteins are also significant targets of compounds in *B. rotunda*. 5,6-Dehydrokawain and 2S-pinocembrin stably bind to VEGFR1 and VEGFR2, respectively. Based on its binding position, the two compounds have high potential as inhibitors of the two proteins. VEGFR1 and VEGFR2 are receptors of the VEGF ligand that have a role in angiogenesis. VEGFR1 and VEGFR2 are overexpressed in breast cancer cells [85, 86]. Previous studies have shown that the inhibition of these two receptors not only inhibits angiogenesis but also cell growth and induces cancer cell apoptosis. Inhibition of VEGFR1 can inhibit angiogenesis in mouse models of breast cancer and decrease the viability of various breast cancer cell lines such as CAL-120, JIMT-1, MCF-7, and MDA-MB-134 [87]. Another study stated that the inhibition of VEGFR1 and VEGFR2 could inhibit growth and induce apoptosis of cancer cells through regulation of the PI3K/Akt and MAPK pathways [88]. Inhibition of VEGF signaling reduces pPI3K and pAKT, which are important proteins in the PI3K/Akt signaling pathway, which causes cells to undergo apoptosis [89]. The inhibition of VEGFR1/2 from *in silico* studies that resulted in decreased cell viability and induction of apoptosis was confirmed by *in vitro* results (Figures 7(c)–7(e))

Cyclin-dependent kinase 4 (CDK4) is a protein that plays an important role in cell cycle regulation. The docking and MD results indicated that cardamonin interacted stably at the abemaciclib binding site of CDK4. This similar interaction with the control indicates cardamonin has potential as a CDK4 inhibitor [61]. In general, cyclin D is overexpressed in breast cancer cells, but it requires CDK4 to perform its function, namely, as a cell cycle regulator [90]. When the CDK4-cyclin D complex is activated, the complex phosphorylates retinoblastoma (RB), which makes RB released from the E2F transcription factor, and then, E2F binds to DNA and initiates transcription of genes needed to enter the S phase [91]. Therefore, CDK4 inhibition can cause cell cycle arrest in the G1 phase [92]. In addition, CDK4 inhibition can also cause cancer cell apoptosis. Previous studies have shown that CDK4 inhibition can reduce NF-kB activity, resulting in downregulation of antiapoptotic genes [93]. This mechanism may also occur in T47D cells treated with *B. rotunda* extract, but further research is needed.

The JAK/STAT pathway has a crucial role in the development and progression of breast cancer [12]. This study showed that there were compounds bound to JAK3 and STAT3 that are predicted to inhibit the activity of these two proteins. The lowest binding affinity value is in the interaction between alpinetin and JAK3. The MD results also show that the interaction between the two is stable. Therefore, alpinetin in *B. rotunda* has high potential as a JAK3 inhibitor. JAK3 activated by receptor tyrosine kinase will activate STAT3, and then, STAT3 forms a dimer and translocates to the nucleus to become a transcription factor related to cell proliferation and survival [75]. Therefore, inhibition of this pathway can induce cancer cell apoptosis. Previous research stated that JAK and STAT inhibition resulted in the apoptosis of MCF-7 breast cancer cells [94].

The anticancer effect of *B. rotunda* predicted by the *in silico* approach was confirmed by the *in vitro* approach. The ethanol extract of *B. rotunda* has an IC_50_ value of 40.4 *μ*g/mL for T47D and 292.7 *μ*g/mL for TIG-1. The result indicates that *B. rotunda* has potential for selective killing between cancer (T47D) and normal (TIG-1) cells. The effect of inducing apoptosis in *B. rotunda* ethanol extract on T47D cells was measured by the rhodamine 123 and apoptosis (annexin V/PI) assays. These results are in line with those of the *in silico* method in which the compounds in *B. rotunda* can inhibit the activity of proteins related to cell survival and antiapoptosis. The apoptotic effect of the *B. rotunda* ethanol extract was presented in the rhodamine 123 assay and the apoptosis assay. The results showed that the cells lost their mitochondrial membrane potential (MMP) when treated with *B. rotunda* ethanol extract. Loss of MMP is an important step in inducing apoptosis because it can facilitate cytochrome c exit from the mitochondria and activate apoptotic signaling [95]. The decrease in MMP was predicted due to the inhibition of KCNA3 activity by pinostrobin chalcone. KCNA3 is a mitochondrial ion channel that controls the mitochondrial membrane potential [96]. Apoptosis of T47D cells due to *B. rotunda* extract was also demonstrated in this study (Figures 7(e) and 7(g)). The apoptosis of T47D is predicted to be due to seven bioactive compounds from *B. rotunda* that interact with breast cancer-related proteins. However, this study shows that several compounds have the potential for antiangiogenesis and antitumor invasion. Further studies are needed on the antiangiogenesis and anti-invasion effects of *B. rotunda* extract on breast cancer cells.

## 5. Conclusion

This study focused on predicting the potential anticancer mechanism of *B. rotunda* against breast cancer cell line using an *in silico* approach. *B. rotunda* contains seven compounds that are predicted to have anticancer effects: sakuranetin, cardamonin, alpinetin, 2S-pinocembrin 7.4′-dihydroxy-5-methoxyflavanone, 5.6-dehydrokawain, and pinostrobin chalcone. These compounds are predicted to stably interact with the MMP12, CDK4, JAK3, VEGFR2, MMP13, VEGFR1, and KCNA3 proteins, which have a role in inhibiting the growth and inducing apoptosis in breast cancer cells. The predicted anticancer activity results were confirmed by in vitro assays where *B. rotunda* extract was shown to be toxic and induce apoptosis of T47D cells. However, further experimental studies are needed to support these findings. This study provides an important basis for further research, considering that the *in silico* results predicted that the compounds in *B. rotunda* targeted multiple pathways related to breast cancer progression.

## Figures and Tables

**Table 1:** Bioactive compounds in *B. rotunda* from the KNApSAcK database and the previous study.

No	Compound	Formula	MW (g/mol)	PubChem ID	Method	Ref
1	(E)-Geraniol	C_10_H_18_O	154.25	637566	HPLC	[53]
2	Sakuranetin	C_16_H_14_O_5_	286.28	73571	NMR	[54]
3	Isopimaric acid	C_20_H_30_O_2_	302.5	442048	XRD	[55]
4	Cardamonin	C_16_H_14_O_4_	270.28	641785	HPLC	[56]
5	Flavokawin A	C_18_H_20_O_5_	316.3	270057	TLC	[57]
6	Boesenbergin A	C_26_H_28_O_4_	404.5	6313827	NMR	[58]
7	Rubranine	C_25_H_26_O_4_	390.5	42607681	NMR	[58]
8	Panduratin A	C_26_H_30_O_4_	406.5	6483648	HPLC	[56]
9	Alpinetin	C_16_H_14_O_4_	270.28	154279	HPLC	[56]
10	2S-Pinocembrin	C_15_H_12_O_4_	256.25	68071	HPLC	[56]
11	5,7-Dihydroxy-8-C-geranylflavanone	C_25_H_28_O_4_	392.5	11143678	HPLC	[53]
12	7,4′-Dihydroxy-5-methoxyflavanone	C_16_H_14_O_5_	286.28	188424	HPLC	[53]
13	(-)-4-Hydroxypanduratin A	C_25_H_28_O_4_	392.5	636530	HPLC	[56]
14	Isopanduratin A	C_26_H_30_O_4_	406.5	10069916	HPLC	[53]
15	2,4-Dihydroxy-6-phenethyl-benzoic acid methyl ester	C_16_H_16_O_4_	272.29	14195786	HPLC	[53]
16	5,6-Dehydrokawain	C_14_H_12_O_3_	228.24	5273621	HPLC	[53]
17	7-Methoxy-5-hydroxy-8-geranylflavanone	C_26_H_30_O_4_	406.5	129864052	HPLC	[53]
18	(+)-Zeylenol	C_21_H_20_O_7_	384.4	14283260	X-ray crystallography	[55]
19	Crotepoxide	C_18_H_18_O_8_	362.3	161314	Spectra, MS, 2D-NMR	[57]
20	Pinostrobin chalcone	C_16_H_14_O_4_	270.2	5316793	HPLC	[56]

**Table 2:** Molecular docking simulation results.

Compound	Control		Protein target		Binding affinity (kcal/mol)	
	Compound	PDB ID/PubChem ID	Protein	PDB ID	PB^a^	PC^b^
Sakuranetin	Tamoxifen	3ert	ERa	3ert	−7.6	−9.5
	Estradiol	5toa	ERb	5toa	−7.6	−11.1
	LP168^*∗*^	6enm^*∗*^	MMP12^*∗*^	6enm^*∗*^	−9.3^*∗*^	−10.4^*∗*^
	RC-3-96	3sqq	CDK2	3sqq	−8.5	−8.7

Cardamonin	Abemaciclib^*∗*^	2w9z^*∗*^	CDK4^*∗*^	2w9z^*∗*^	−8.2^*∗*^	−8.5^*∗*^
	Estradiol	5toa	ERb	5toa	−7.6	−11.1
	Wortmannin	1e7u	PI3K	1e7u	−7.5	−9
	Dacomitinib	4i23	EGFR1	4i23	−7.5	−8.2

Alpinetin	Tamoxifen	3ert	ERa	3ert	−7.7	−9.5
	Estradiol	5toa	ERb	5toa	−7.5	−11.1
	Quinoxaline	3krr	JAK2	3krr	−8.3	−11.5
	Pyrrolopyridazine carboxamide^*∗*^	6ny4^*∗*^	JAK3^*∗*^	6ny4^*∗*^	−8.3^*∗*^	−8.2^*∗*^
	Inhibitor	59239114	mTOR	4jsv	−7.6	−5.1

2S-Pinocembrin	Tamoxifen	3ert	ERa	3ert	−8.6	−9.5
	Estradiol	5toa	ERb	5toa	−8.7	−11.1
	Inhibitor	2ow9	MMP13	2ow9	−9.6	−11.6
	LP168	6enm	MMP12	6enm	−9.3	−10.4
	Pyrrolopyrimidine^*∗*^	3vhe^*∗*^	VEGFR2^*∗*^	3vhe^*∗*^	−9.8^*∗*^	−12.9^*∗*^

7.4′-Dihydroxy-5-methoxyflavanone	Tamoxifen	3ert	ERa	3ert	−7.5	−9.5
	Estradiol	5toa	ERb	5toa	−8.1	−11.1
	Inhibitor^*∗*^	2ow9^*∗*^	MMP13^*∗*^	2ow9^*∗*^	−9.3^*∗*^	−11.6^*∗*^
	Dinaciclib	6gu6	CDK1	6gu6	−8.2	−8.7
	Hydroxy pioglitazone	A6dha	PPARG	6dha	−8.8	−9.4

5.6-Dehydrokawain	Inhibitor	16097729	EGFR1	4i23	−7.2	−8.2
	Pyrrolopyridazine carboxamide	6ny4	JAK3	6ny4	−7.5	−8.2
	Inhibitor^*∗*^	16097729^*∗*^	VEGFR1^*∗*^	3hng^*∗*^	−8.4^*∗*^	−10.6^*∗*^
	Inhibitor	3pze	MAPK8	3pze	−6.8	−7.4
	SI109	6nuq	STAT3	6nuq	−5.3	−9.2

Pinostrobin chalcone	Inhibitor	16097729	EGFR1	4i23	−6.9	−8.2
	Pyrazoloquinazoline	2xch	PDPK1	6nuq	−7.1	−9.1
	Inhibitor^*∗*^	247938^*∗*^	KCNA3^*∗*^	7ej1^*∗*^	−8.3^*∗*^	−8.1^*∗*^
	A-769662	4cff	AMPK	4cff	−7.5	−10

PB^a^: binding energy compound from *B. rotunda* protein; PC^b^: control protein; ^*∗*^the protein that has the most negative binding affinity when interacting with compounds in *B. rotunda*.

## Data Availability

The datasets used and analyzed during the present study are available from the corresponding author on reasonable request.
